# The Week's Work

**Published:** 1910-04-09

**Authors:** 


### The Week's Work.

### THE WEIR CHARITY.

The judgment of Mr. Justice Eve, delivered on the 17th of last month, has for the present put an end to the disputes which have arisen in connection with the administration of this charity, though we understand that an appeal is to be entered against the learned judge's decision. The testator, Mr. Benjamin Weir, who died in February 1902, devised two freehold houses to trustees upon trust to use one as a dispensary, cottage hospital, convalescent home, or other medical charity (to be called the "Weir Hospital") for the benefit of the inhabitants of Streatham and the neighbourhood; and to use the other in conjunction therewith on the expiration of certain restrictive covenants. The residue of his real and personal property the testator left for the support and maintenance of the hospital, but a limit of £400 a year was placed on the trustees' expenditure so long as the hospital was carried on in the first house only; the balance being invested in order to provide a fund for the necessary alterations in the second house to adapt it for the purposes of a hospital and the erection of additional buildings. Provision was also made for the creation of a committee of management, consisting of the trustees, with others nominated by the Streatham Vestry (whose successors are the Corporation of the Borough of Wandsworth) or co-opted by the other members of the committee. The gross income of the moneys at the disposal of the trustees was over £3,000.

### A SCHEME TO REGULATE CHARITY.

Application was made by a majority of the trustees to the Charity Commissioners for a scheme to regulate the charity, and subject to the approval of the Commissioners and the preparation of a scheme, an agreement was entered into with the committee of the Bolingbroke Hospital, Battersea, whereby a very large sum was to be applied for the purposes of that hospital. Strong local opposition was raised to this proposal, and a public inquiry was held by the Charity Commissioners into the matter; but eventually a scheme was drafted which in effect combined the Weir Charity with the Bolingbroke Hospital, which was in future to be known as the Weir and Bolingbroke Hospital. One of the houses devised by the testator was to be used as a dispensary (with local trustees), to be called the "Weir Dispensary," for the benefit of Streatham and the neighbourhood, and £400 a year was to be devoted to its support; the other house was to be used as a Nurses' Home in connection with the Weir and Bolingbroke Hospital; and £350 a year was to be handed over to the local trustees to be used at their discretion for what may be called medical and surgical purposes. £50,000 of the Weir Charity was to go to the hospital, in which patients from Streatham and the neighbourhood were to have certain preferential rights. This scheme was finally sealed in December 1908.

### THE WANDSWORTH PETITION.

The Corporation of the Borough of Wandsworth thereupon petitioned the Court that the scheme might be set aside or remitted for further consideration. It was argued that it deviated unnecessarily from the express terms of the trust and the wishes of the testator, who (it was said) obviously desired to found a hospital in Streatham and not to benefit an institution in Battersea. The establishment of the Nurses' Home was especially attacked as not being a "medical charity" at all. It was also said that the existence of a large surplus in the hands of the trustees did not of itself warrant an application of the *cy-près* doctrine by the Charity Commissioners. Mr. Justice Eve found against the petitioners on all these points. He held that the Nurses' Home was a "medical charity," and few will be disposed to disagree with him in this. He was also of opinion that he could not, in view of many previous decisions of the Courts, review the findings of the Commissioners, and that he must assume that they had satisfied themselves that a good case for the application of the *cy-près* doctrine had been made out; and that it was not for him to discuss alternative schemes, however beneficial. The judgment, therefore, turned rather on questions of law than of fact, and in view of the pending appeal it would not be right to comment further on it at present. But the result is the enlargement and improvement of an established institution instead of the creation of a new and necessarily small one, and this is in accordance with the whole trend of modern hospital administration. The Charity Commissioners are to be congratulated on the enlightened view which they took of the situation and on the confirmation of their careful and well-thought-out scheme.

### THE RATING OF HOSPITALS.

The complexity of this very difficult and important subject is well illustrated by the letters, written from several different points of view, which have appeared during the past fortnight in the lay press. The rating of hospitals is a very old question, and a very sore one with some people; like most other large subjects it has many sides to it, and any solution of its problems must take all these into account. Our own views on the subject were definitely declared as long ago as March 15, 1902, when we cordially supported the attempt to obtain exemption for the hospitals, and we have since seen no reason to deviate from this position. In the present instance the ball appears to have been set rolling by Earl Cawdor when presiding at the annual meeting of the Homœopathic Hospital, Great Ormond Street. Commenting on the maintenance of fire brigades, asylums, libraries, police, and other public services out of the rates, his lordship asked why hospitals should be so taxed by municipal authorities that the payment of rates means a very serious strain on their resources. He is reported to have urged that "if hospitals were exempted from municipal taxes, it would go very far to remove the financial difficulties under which many of them laboured." This pronouncement promptly called forth a champion of the opposite camp, a ratepayer of the Parish of St. George the Martyr, in which the hospital is situated. In a letter which was subsequently sent to the press, this gentleman pointed out that the parish contains five hospitals, and that to exempt all these from rates would be to saddle the already overburdened ratepayers with an extra amount of taxation.

### HOSPITAL SECRETARIES' VIEWS.

Following this correspondence the secretaries of three of the hospitals concerned (the Homœopathic, the Great Ormond Street, and the National) contributed their views to the *Evening Standard*; and it is interesting to see their ideas therein expressed. Two of them—Mr. Johnson, of Great Ormond Street, and Mr. Hamilton, of the National—are decidedly against the total exemption of hospitals from local taxation. At the same time they both protest against the excessive incubus beneath which the finances of their respective institutions stagger, owing to the valuable premises which have been erected for the carrying on of their work. It seems that for extending the out-patient department of the Hospital for Sick Children the parish practically fines the hospital £500 a year; for that is the additional sum collected in rates on the revised assessment of the buildings as now added to. The total rates which the hospital pays amount to £1,374 annually, a sum almost equal to the combined grants of the Hospital Sunday and Saturday Funds. It is of this extra burden that Mr. Johnson complains most; for he admits the point that it is unfair on the ratepayers of one borough to expect them to bear the burdens that rightly they should share with many neighbouring and even distant ones. Mr. Hamilton takes a somewhat similar attitude. It seems reasonable, he says, that when a hospital is built or enlarged it should pay rates only on the values it displaces and not on additional values it creates.

### THE LUNACY LAWS.

An energetic campaign would appear to be in progress against the present working of the Lunacy Acts, 1890, and a public meeting of protest is announced to be held in London shortly. Our contemporary, *John Bull*, invites all who consider that they have been unjustly confined or detained in an asylum to communicate to Dr. Forbes Winslow particulars of their cases. We should be loth to give any further publicity to this very mischievous invitation were it not that the editor of *John Bull* may possibly be unaware of the harm that is likely to ensue from it. This is not the first time that the journal in question has indulged in sensation-mongering over the Lunacy Laws, and it is time that a protest is offered. Only a year or two ago, in " scare " headlines and a page or two of flamboyant nonsense, it was announced that an eminent surgeon (who was not named then, and shall be nameless now) had been unjustly and illegally confined in a madhouse by the guilty connivance of his wife and certain physicians. Had the editor taken the trouble to investigate the facts he would speedily have satisfied himself that the gentleman in question, whose identity was easily penetrated, was at one time extremely violent, and actually committed (without the slightest provocation) a most serious assault on one of the doctors in charge of the private asylum to which he had been sent. And he can, if he likes, satisfy himself now that since the surgeon's release his mental condition has been such as amply to justify the original incarceration. Before *John Bull* lends its influence to any campaign conducted by Dr. Winslow, the editor may fairly be asked very seriously to consider for what reasons his paper is being committed to such an unwise course. If he feels that a desire to exploit the public appetite for sensation forms any part of the attractions of the scheme, he should in common honesty withdraw from an agitation which is, in the estimation of the medical profession and of lunacy experts particularly, likely to be highly prejudicial to the real interests of the public.

### THE DRUG MARKET.

A slight improvement in the demand for drugs has been noticeable during the week, and the outlook seems more promising. Several price alterations have been made, at least one of which was not generally expected—namely, the alteration in the price of codeine, which has been reduced by 5d. per ounce. Morphine is unchanged in price, and the value of opium is well maintained, reports from the East indicating that no substantial reduction in the price of opium is to be expected for six weeks at least. Saffron is advancing in price. Ipecacuanha shows no signs of becoming cheaper at present. Chrysarobin is 6d. per lb. dearer. English refined camphor is 1d. per lb. lower. The price of acetyl-salicylic acid has been advanced 3½d. per lb., and it is not altogether improbable that a further advance may be made before long, but this is, of course, uncertain. The most important price-alteration is a reduction of 9d. per oz. in makers' quotations for cocaine. The demand for cod-liver oil is becoming very quiet, and there seems little reason to expect any material advance in value.

